# Home-site advantage for host species–specific gut microbiota

**DOI:** 10.1126/sciadv.adf5499

**Published:** 2023-05-12

**Authors:** Daniel D. Sprockett, Jeffrey D. Price, Anthony F. Juritsch, Robert J. Schmaltz, Madalena V. F. Real, Samantha L. Goldman, Michael Sheehan, Amanda E. Ramer-Tait, Andrew H. Moeller

**Affiliations:** ^1^ Department of Ecology and Evolutionary Biology, Cornell University, Ithaca, NY, USA.; ^2^ Department of Food Science and Technology, University of Nebraska-Lincoln, Lincoln, NE, USA.; ^3^ Nebraska Food for Health Center, University of Nebraska-Lincoln, Lincoln, NE, USA.; ^4^ Laboratory for Animal Social Evolution and Recognition, Department of Neurobiology and Behavior, Cornell University, Ithaca, NY, USA.

## Abstract

Mammalian species harbor compositionally distinct gut microbial communities, but the mechanisms that maintain specificity of symbionts to host species remain unclear. Here, we show that natural selection within house mice (*Mus musculus domesticus*) drives deterministic assembly of the house-mouse gut microbiota from mixtures of native and non-native microbiotas. Competing microbiotas from wild-derived lines of house mice and other mouse species (*Mus* and *Peromyscus* spp.) within germ-free wild-type (WT) and *Rag1*-knockout (*Rag1*^−/−^) house mice revealed widespread fitness advantages for native gut bacteria. Native bacterial lineages significantly outcompeted non-native lineages in both WT and *Rag1*^−/−^ mice, indicating home-site advantage for native microbiota independent of host adaptive immunity. However, a minority of native Bacteriodetes and Firmicutes favored by selection in WT hosts were not favored or disfavored in *Rag1*^−/−^ hosts, indicating that *Rag1* mediates fitness advantages of these strains. This study demonstrates home-site advantage for native gut bacteria, consistent with local adaptation of gut microbiota to their mammalian species.

## Teaser

Adaptive advantages for native bacteria underlie assembly of the house-mouse gut microbiota.

## INTRODUCTION

The gut microbial communities of diverse mammalian species reflect the evolutionary histories of their hosts. In rodents, xenarthrans, artiodactyls, primates, and other mammalian clades, hosts of the same species (i.e., conspecifics) tend to harbor more similar gut microbiota compositions than do hosts of different species, and microbiota dissimilarity between host species is positively associated with the hosts’ evolutionary divergence (*1*–*7*). Experimental work in rodents has indicated that disruption of host species–specific microbiotas can have adverse consequences for hosts, including impaired resistance to pathogen colonization (*8*), diminished nutrient utilization (*9*), and reduced growth rate (*10*). Nevertheless, the ecological and evolutionary forces underlying microbiota specificity to host species have not been determined (*11*–*14*).

One proposed mechanism is biased microbial dispersal (i.e., dispersal limitation). Microbial dispersal among conspecifics occurs readily through both social (*15*–*18*) and vertical transmission (*19*–*23*), whereas dispersal between host species tends to be less frequent (*3*, *24*). The bias toward dispersal among conspecifics could promote and maintain microbiota divergence between host species in the absence of selective processes (e.g., through ecological drift) (*3*, *13*). A nonmutually exclusive mechanism is adaptation of symbionts to their respective host-associated environments (i.e., local adaptation). For example, previous experiments have shown that strains of the gut bacterium *Lactobacillus reuteri* derived from house mice (*Mus musculus domesticus*) display higher fitness within house mice than do *L. reuteri* strains from other mammalian host species (*25*–*27*), indicating home-site advantage for native *L. reuteri* consistent with local adaptation. However, the extent to which constituents of host species–specific microbiotas are favored by natural selection within their host species remains unclear. Quantifying the relative influences of these alternative mechanisms—dispersal limitation and local adaptation—remains a critical gap in understanding the assembly of host species–specific microbiota in mammals.

## RESULTS AND DISCUSSION

**Fig. 1.**
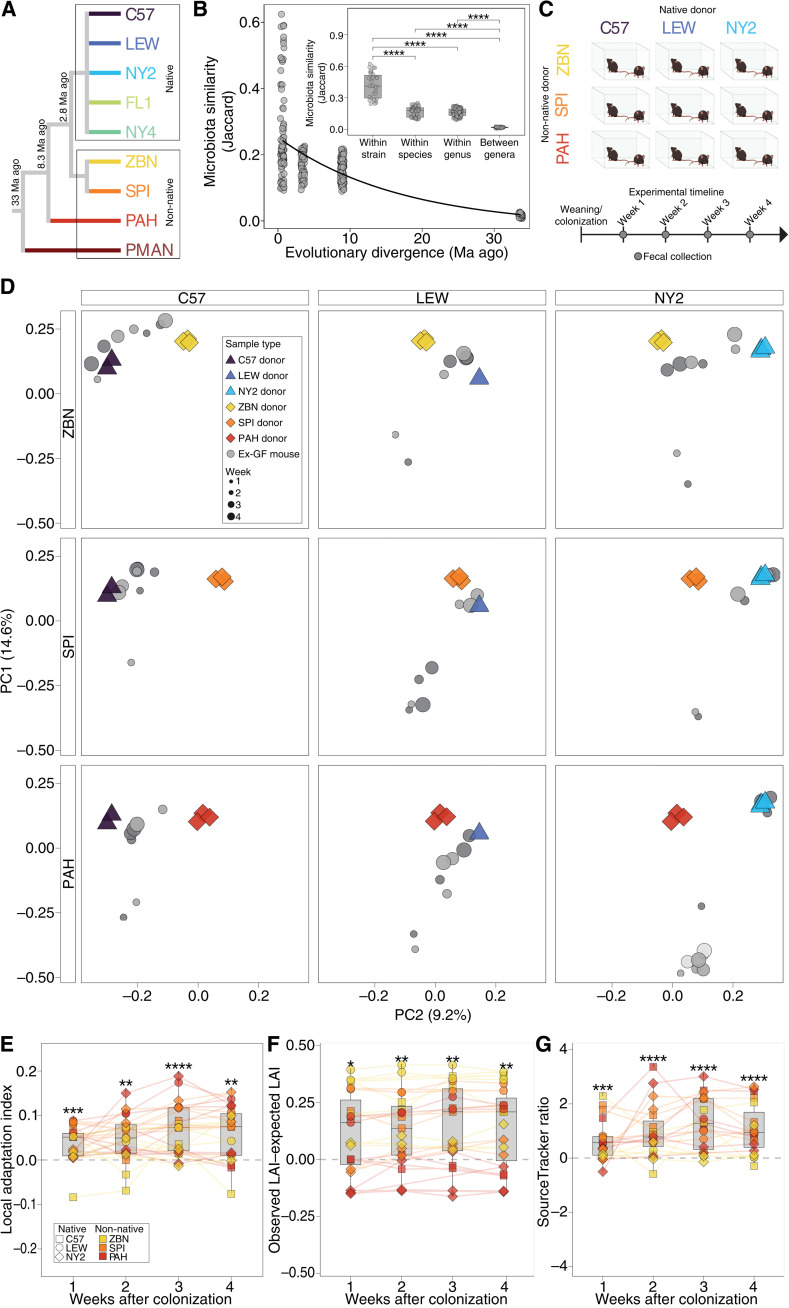
Deterministic assembly of house-mouse microbiota from mixtures of native and non-native microbiotas. (**A**) Phylogeny shows evolutionary relationships among wild-derived laboratory mice from which microbiotas were obtained. (**B**) Scatterplot shows negative association between microbiota similarity (Jaccard) and evolutionary divergence [millions of years ago (Ma ago)] among rodent hosts. Divergence times were estimated using data from TimeTree.org. Curve shows exponential decay regression (*P* < 0.001, *R*^2^ = 0.54). Inset shows boxplots of microbiota similarity between pairs of samples. Wilcoxon test, FDR-adjusted ****P < 1 × 10^−4^. (**C**) Cartoon shows experimental design. Fecal microbiotas from three native *M. m. domesticus* lines and three non-*domesticus* mouse lines were mixed in pairwise combinations and inoculated into weaned germ-free mice, from which fecal samples were collected weekly for 4 weeks. (**D**) PCoA plots show similarities among microbiotas from donors and ex-germ-free recipients based on the Jaccard similarity index. Colors indicate the mouse line from which the samples were collected corresponding to (A). Sizes of gray circles indicate time points (weeks 1 to 4), and shades of gray delineate host individuals. The PCoA plot is faceted by inoculum but plotted along common axes showing microbiota similarity among all donors and recipient ex-germ-free mice. (**E**) Boxplots show positive LAI values of ex-germ-free mice throughout the 4-week experiment; (**F**) positive differences between observed LAI values and LAI values expected under neutral assembly; and (**G**) log_10_-transformed ratios of native ASVs to non-native ASVs identified as sources by SourceTracker. In (E) to (G), shapes and colors denote identities of native and non-native donors, respectively, and lines connect samples from the same mouse. For each boxplot in (B) and (E) to (G), center lines denote medians, and lower and upper hinges correspond to first and third quartiles, respectively. FDR-adjusted *P* values were derived from Wilcoxon tests for non-zero mean, *P < 0.05, **P < 0.01, ***P < 0.001, and ****P < 0.0001.

We tested for home-site advantage of the house-mouse gut microbiota relative to gut microbiotas from closely related mouse species through a series of in vivo microbiota competition experiments. First, we characterized the gut microbiotas of nine laboratory mouse lines of house mice and other non-*domesticus* mouse species. These included the house-mouse model C57BL/6 and eight wild-derived lines of three species within the genus *Mus* (*M. m. domesticus*, *Mus spicilegus*, and *Mus pahari*) and one species of deer mouse (*Peromysucs maniculatus*) (Fig. 1A and table S1), all of which represent omnivore generalists. Wild-derived mouse lines were maintained under laboratory conditions for >10 host generations and descended directly from mice collected in the wild (i.e., they were never rederived through embryo transplantation or cross-fostering with a laboratory mouse line) to facilitate the retention of host-lineage specific microbiota (*10*, *23*). Moreover, these wild-derived lines never received antibiotics. Amplicon sequencing of the 16*S* ribosomal RNA (rRNA) gene from fecal samples indicated that host species maintained compositionally distinct microbiotas in the laboratory environment [fig. S1, A and B; permutational multivariate analysis of variance (PERMANOVA), *P* < 0.001]. Furthermore, microbiota similarity between hosts decayed exponentially as a function of host evolutionary divergence time (Fig. 1B; *R*^2^ = 0.54, *P* < 0.001), recapitulating what has been observed in wild rodent species (*3*). Mean microbiota similarity between individual hosts was highest within host lines, followed by between line/within species, between species, and between genera (Fig. 1B, inset). A significant negative relationship between microbiota similarity and host relatedness was also observed within the genus *Mus* alone (fig. S2; *R*^2^ = 0.19, *P* < 0.001) and when only interline comparisons were considered (*R*^2^ = 0.12, *P* < 0.001). Together, these results indicate that the laboratory mouse lines have maintained host species–specific microbiotas associated with their hosts’ phylogenetic histories.

Using samples from the diverse set of mice, we then conducted competition experiments in which microbiotas from house mice (i.e., native microbiota) and from non-*domesticus* mouse species (i.e., non-native microbiota) were coinoculated at defined ratios into germ-free house mice. The first set of experiments tested nine distinct pairwise mixtures of native and non-native microbiotas derived from three of the *M. m. domesticus* mouse lines (i.e., native donors: C57BL/6, LEWES, and NY2) and three non-*domesticus* mouse lines (i.e., non-native donors: *M. spicilegus* ZBN and SPI/TUA and *M. pahari* PAH) (Fig. 1C). Pairs of native and non-native fecal samples from sex- and age-matched mice were mixed equally by weight (~25 mg of feces from each donor), and each mixture was inoculated at weaning into two germ-free *M. m. domesticus* (C57BL/6) hosts reared together in an individual microisolator cage (fig. S3A). Fecal samples from ex-germ-free recipient mice were collected weekly for 4 weeks and profiled using 16*S* rRNA gene amplicon sequencing. Microbiota similarity (Jaccard) between ex-germ-free mice at week 1 and their corresponding donor fecal samples was significantly higher on average than was that between the ex-germ-free mice and fecal samples from other donors based on analyses of both individual recipients [fig. S4; Wilcoxon test, false discovery rate (FDR)–adjusted *P* < 1 × 10^−5^] and recipient cage mean microbiota compositions (Wilcoxon test, FDR-adjusted *P* < 1 × 10^−5^), indicating successful inoculations. The increased microbiota similarity to donors was also evident in a principal coordinates analysis (PCoA) (Fig. 1D).

To test for home-site advantage of native microbiota, we calculated microbiota similarity between ex-germ-free mice and their native or non-native donors. We defined the difference between the microbiota similarity to the native donor and the microbiota similarity to the non-native donor as the local adaptation index (LAI) (fig. S3B). Mean LAI values were significantly greater than zero for ex-germ-free mouse microbiotas at every time point (Fig. 1E, FDR-adjusted *P* < 0.05, and table S2), indicating that microbiota similarity to native donors was higher than that to non-native donors. Positive LAI values of ex-germ-free mouse microbiotas are consistent with home-site advantage for native microbiotas relative to non-native microbiotas. However, differences in bacterial load between donor fecal samples could lead to differences in colonization success between native and non-native microbiotas in the absence of local adaptation or home-site advantage. Moreover, positive LAI could result from differences in alpha diversity between the native and non-native microbiotas included in the mixtures, even if the microbiotas colonized ex-germ-free house mice equally well. To address these potential issues, we calculated the expected LAI for each ex-germ-free mouse microbiota under a neutral model of community assembly (*28*), given the measured microbiota composition and bacterial load (i.e., 16*S* rRNA gene copy number) of the two donor fecal samples included in the mixture that the mouse received (table S3). The difference between the observed LAI and the expected LAI provided a test statistic for the degree of competitive advantage of the native microbiota over the non-native microbiota (fig. S3C and table S2). Results showed that native microbiotas were selected over non-native microbiotas in ex-germ-free house mice throughout the 4-week experiments beyond what would be expected under neutrality (Fig. 1F; Wilcoxon test, FDR-adjusted *P* < 0.05 for all comparisons; observed LAI–expected LAI > 0). In addition, parallel analyses based on SourceTracker (*29*) indicated that ex-germ-free mouse microbiotas were composed of a significantly greater microbiota fraction derived from the native donor than from the non-native donor (Fig. 1G; Wilcoxon test, FDR-adjusted *P* < 0.001 for all comparisons) (see the Supplementary Materials). Significantly positive LAI, observed LAI–expected LAI, and SourceTracker ratio values were also observed on the basis of analyses of weighted β-diversity measures (Bray-Curtis) (Wilcoxon test, FDR-adjusted *P* < 0.01 for all comparisons). A minority of ex-germ-free recipients displayed increased microbiota similarity to non-native donors relative to native donors based on individual measures of home-site advantage (Fig. 1, E to G); however, on average, we observed significant competitive advantages for native microbiota in these experiments. Cumulatively, these results show that, when competed within germ-free house mice, native house-mouse microbiotas significantly outcompeted non-native microbiotas from non-*domesticus* host species.

**Fig. 2.**
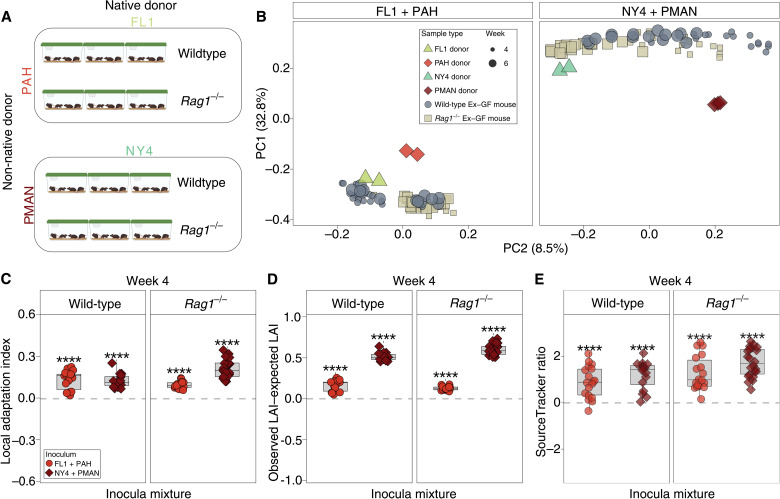
Competitive advantages of native microbiota in both WT and *Rag1*^−/−^ mice. (**A**) Cartoons show experimental design. Fecal samples from two *M. m. domesticus* lines and two non-*domesticus* mouse strains were mixed in equal ratios and inoculated into germ-free mouse pups at 10 days of age reared in sterile, multicage gnotobiotic isolators. Fecal samples were collected at 4 and 6 weeks. (**B**) PCoA plots show microbiota similarities (Jaccard) among donors and ex-germ-free recipients. Colors indicate mouse lines from which samples were collected. Sizes of gray circles indicate time points (weeks 4 and 6). As in Fig. 1, the PCoA plot is faceted by inoculum but plotted along common axes showing microbiota similarity among all donors and recipient ex-germ-free mice. (**C** to **E**) Boxplots show LAI values (C), the differences between observed and expected LAI values (D), and log_10_-transformed ratios of native ASVs to non-native ASVs identified as sources by SourceTracker (E). For (C) to (E), shapes and colors denote identities of native and non-native donors, respectively. Wilcoxon test for non-zero mean, FDR-adjusted ****P < 0.0001. For each boxplot in (B) to (D), center lines denote medians, and lower and upper hinges correspond to first and third quartiles, respectively.

Next, we tested whether competitive advantages for native microbiotas over non-native microbiotas depend on the presence of the host adaptive immune system. Adaptive immunity in mammals is both a highly specific microbial filter and a facilitator of colonization for certain microbiota constituents (*30*–*32*). To assess the degree to which host adaptive immunity contributes to selection for host species–specific microbiota, we conducted additional microbiota competition experiments in which two mixtures of native and non-native microbiotas—*M. m. domesticus* (FL1) + *M. pahari* (PAH) and *M. m. domesticus* (NY4) + *Peromyscus maniculatus* (PMAN)—were inoculated into wild-type (WT) germ-free C57BL/6 mice (*n* = 34) and a line of germ-free C57BL/6 in which *Rag1* gene has been deleted (*Rag1^−/−^*) (*n* = 40) (Fig. 2A). These pairs of donors were selected because they represented the greatest degrees of evolutionary divergence between donors, including divergence between the genera *Mus* and *Peromyscus*. *Rag1^−/−^* mice lack mature T and B cells and a functional adaptive immune system (*33*), and deletion of *Rag1* has previously been shown to alter the microbiota relative to WT hosts (*30*, *31*, *34*, *35*). In contrast to the first set of experiments (Fig. 1), which assessed the consistency across biological replicates of native and non-native donor combinations the degree to which *M. m. domesticus* microbiotas displayed home-site advantage, this second set of experiments was designed to provide sufficient technical replication to enable quantification of the effect of host genotype (i.e., WT versus *Rag1^−/−^*) on the degree of home-site advantage for *M. m. domesticus* microbiota. To address this question, each microbiota mixture was gavaged into mice residing in a single, sterile isolator containing five to seven cages and two to four mice per cage. Mice of the same sex and genotype were cohoused. Fecal samples from ex-germ-free mice were collected at 4 and 6 weeks after inoculation, and microbiota profiles were generated with 16*S* rRNA gene sequencing. As in the first set of experiments, microbiota similarity (Jaccard) between the ex-germ-free mice and the donor fecal samples that they received was significantly higher, on average, than that between the ex-germ-free mice and other donor fecal samples. These results were observed on the basis of analyses of individual recipient mice (Fig. 2B and fig. S5; Wilcoxon test, FDR-adjusted *P* < 1 × 10^−5^) and recipient cage mean microbiota compositions (Wilcoxon test, FDR-adjusted *P* < 1 × 10^−5^). The microbiota similarity of recipients to their donors in this second set of experiments was higher than that observed for the first set of experiments (fig. S4), potentially due to differences in animal husbandry techniques used (e.g., multicage isolators versus single-cage isolators). These results indicate successful colonization of the microbiota mixtures after gavage.

This second set of experiments revealed reproducible assembly of the house-mouse gut microbiota from mixtures of native and non-native microbiotas regardless of the presence or absence of a functional adaptive immune system in hosts. α- and β-diversity differed between ex-germ-free WT and *Rag1*^−/−^ mice (Fig. 2B, PERMANOVA, *P* < 0.001, recipient genotype: *R*^2^ = 0.1 and inoculum type: *R*^2^ = 0.06; and fig. S6, Wilcoxon test, *P* < 0.05 for both inocula), but significant evidence for local adaptation was observed in both host genotypes. The effect sizes of host genotype on microbiota composition (*R*^2^ = 0.1) were in line with previous reports of the effect of immune gene knockouts (including *Rag1*, *MyD88*, and *Nod2*) on microbiota ranging from *R*^2^ = 0.08 to 0.23 (*36*). LAI values of ex-germ-free mice were positive at both at 4 weeks (Fig. 2C and table S4) and 6 weeks (fig. S7A and table S4) (Wilcoxon test, FDR-adjusted *P* < 0.001 for all comparisons). Moreover, in both experiments and both host genotypes, the observed LAI was significantly greater than expected LAI (Fig. 2D, fig. S7B, and table S4; Wilcoxon tests, FDR-adjusted *P* < 0.001 for all comparisons; observed LAI–expected LAI > 0), and microbiotas of ex-germ-free mice contained a greater microbiota fraction originating from the native donor than from the non-native donor (based on SourceTracker) (Fig. 2E and fig. S7C; Wilcoxon tests, FDR-adjusted *P* < 0.001 for all comparisons). Significantly positive LAI, observed LAI–expected LAI, and SourceTracker ratio values were also observed on the basis of analyses of weighted β-diversity measures (Bray-Curtis) (Wilcoxon test, FDR-adjusted *P* < 0.01 for all comparisons). Each of these values was significantly higher in *Rag1*^−/−^ mice that received the NY4 + PMAN inoculum than those in WT mice that received this inoculum (Wilcoxon test, FDR-adjusted *P* < 0.001 for all comparisons), suggesting that, in some cases, host adaptive immunity can reduce the competitive advantage of native microbiota over non-native microbiota. These results confirm home-site advantage for house-mouse gut microbiota within house mice and, furthermore, demonstrate competitive advantages for native microbiota independent of host adaptive immunity.

One possible explanation for the observed competitive advantages of house-mouse microbiotas over non-native microbiotas is that house-mouse microbiotas may be superior colonizers regardless of host environment or that non-native microbiotas may be fundamentally unable to colonize germ-free house mice. However, additional experiments in which microbiotas from mouse lines were inoculated individually into germ-free C57BL/6 J revealed no consistent difference in colonization success between native and non-native microbiotas. Microbiotas from mice colonized by native microbiotas were not significantly more similar to the microbiotas of their donors than were those from mice colonized by non-native microbiotas, indicating that similar fractions of the microbiota were able to colonize the germ-free recipient from both native and non-native donors (fig. S8B). Furthermore, microbiotas from mice colonized by either native or non-native microbiotas displayed similar α-diversity and microbe load (fig. S8, C and D). Hundreds of non-native amplicon sequence variants (ASVs) were detected in individually inoculated ex-germ-free house mice and their corresponding non-*domesticus* donor but not detected in ex-germ-free house mice that received fecal mixtures containing samples from the non-*domesticus* donor (see the “Singly colonized mouse experiments” section in the Supplementary Materials and data file S1). Moreover, we recalculated LAI values for ex-germ-free mice from the first set of experiments (Fig. 1) using the microbiotas of ex-germ-free house mice inoculated with individual microbiota as the reference native or non-native donors, based on the microbiota that the ex-germ-free mice inoculated with a single microbiota received. These analyses also revealed significantly positive LAI values across all time points (fig. S9), indicating that, even when only the ASVs that were observed to colonize germ-free house mice gavaged with a single microbiota were considered, native microbiota significantly outcompeted non-native microbiota within germ-free house mice. Thus, house mice represent potential niche space (*37*) for many of the gut bacterial lineages from non-*domesticus* mouse species; however, these non-native bacteria tend to be excluded when inoculated in a competitive context with gut bacteria derived from house mice.

Because germ-free C57BL/6 J mice were used as recipients in all microbiota competition experiments, we conducted an additional set of microbiota-competition experiments to assess whether C57BL/6 J microbiota outcompeted wild-derived microbiota from other house-mouse lines. These experiments were conducted with the same procedures but, instead, considered C57BL/6 J as the native donor and either LEWES and NY2 as the “non-native” donor. In these experiments, C57BL/6 J microbiota displayed a nonsignificant trend of fitness advantages over microbiotas from wild-derived donors (fig. S10). We note that, compared to the first set of experiments (Fig. 1), these experiments were conducted with a relatively small number of biological replicates, providing limited power to detect significant differences. These results do not contradict the observations that wild-derived house-mouse microbiotas outcompeted wild-derived microbiotas from other mouse species, a pattern indicative of home-site advantage and local adaptation of the house-mouse microbiota. Rather, these results suggest that the competitive advantages for native microbiota over non-native microbiota that we observed between host species may extend to differences between microbiotas among host populations within a species. Assessing this possibility will require additional experiments that quantify outcomes of microbiota competition experiments focused pairs of microbiotas from conspecific hosts.

Across all native and non-native donors (Fig. 1A) used in the experiments (Figs. 1 and 2), we identified several ASVs that were consistently differentially abundant between native and non-native donors (fig. S11). Results from these analyses allowed us to test whether these differentially abundant ASVs, for which native or non-native status could be determined with the highest confidence, also showed significant evidence of home-site advantage for native ASVs. To test this prediction, we filtered the ASV table to only the ASVs that were present in three or more native or three or more non-native donors and that displayed the strongest evidence for differential abundance between native and non-native donors (see the Supplementary Materials). Recalculating LAI values for both experiments based on this filtered ASV table, which included only the ASVs that consistently differentiated native and non-native donor lines, also indicated significantly positive LAI values for ex-germ-free mice at every time point (Wilcoxon test, FDR-adjusted *P* < 0.05 for all comparisons) (fig. S11, B and C). These results provide additional support for home-site advantage for native over non-native gut microbiota.

**Fig. 3.**
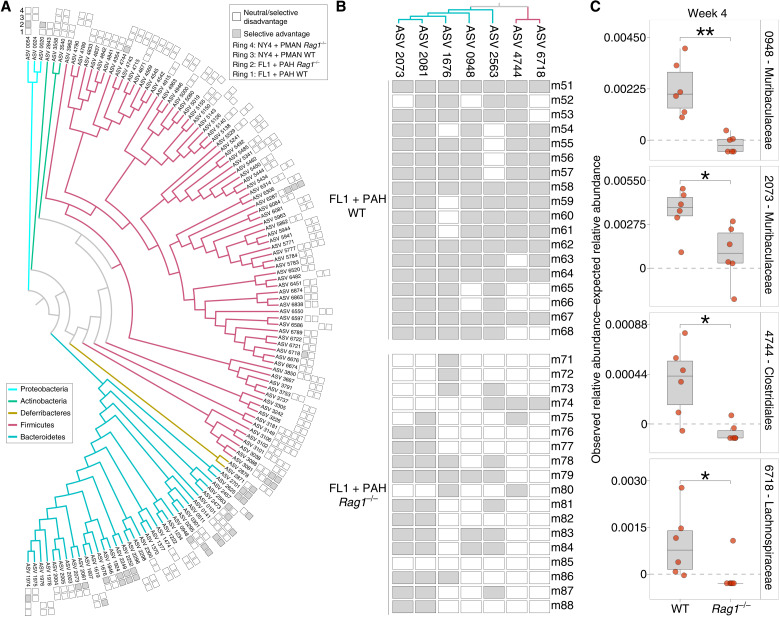
Selective advantages for a subset of native ASVs depended on *Rag1*. (**A**) Phylogeny shows relationships among *M. m. domesticus*–specific ASVs detected in ex-germ-free mice that received the NY4 + PMAN or FL1 + PAH microbiota mixtures. Colors of branches denote bacterial phyla. Rings correspond to ex-germ-free mouse groups (innermost: FL1 + PAH WT; second from innermost: FL1 + PAH *Rag1*^−/−^; second from outermost: NY4 + PMAN WT; and outermost: NY4 + PMAN *Rag1*^−/−^) and indicate significantly positive selection on ASVs (filled squares) within ex-germ-free mice based on binomial tests. Unfilled squares mark ASVs that were detected in ex-germ-free mice but not significantly positively selected. Absence of squares indicates that the ASV was not detected in the mouse group. (**B**) Phylogeny from (A) pruned to only ASVs displaying significant selective advantages in WT ex-germ-free mice but not in *Rag1*^−/−^ mice. Rows correspond to individual ex-germ-free mice, and columns correspond to the tips of the phylogeny. Filled squares indicate ex-germ-free mice in which the observed relative abundance of the ASV exceeded the relative abundance expected under neutrality. (**C**) Boxplots display differences between observed and expected ASV relative abundances in WT and *Rag1*^−/−^ mice that received the FL1 + PAH mixture. All ASVs from both inocula in (B) for which cage-mean differences between host genotypes remained significant after FDR correction are shown. FDR-adjusted *P < 0.05 and **P < 0.01.

Given the widespread evidence of local adaptation of the house-mouse gut microbiota, we next identified the individual gut bacterial lineages that were favored by selection in ex-germ-free house mice. Theses analyses focused on the second set of experiments (Fig. 2), which provided sufficient within-inoculum replication to identify statistically significant evidence for selection on individual ASVs. We calculated the expected relative abundance in ex-germ-free mice for every ASV in the FL1 + PAH and NY4 + PMAN fecal mixtures (Fig. 2A) under a model of neutral assembly based on the compositional profiles and bacterial loads of the donor samples (see the Supplementary Materials). Comparing the observed ASV frequencies with the expected ASV frequencies revealed 33 ASVs that displayed consistent positive deviations from neutrality in WT ex-germ-free mice and 30 ASVs that displayed consistent positive deviations from neutrality in *Rag1*^−/−^ ex-germ-free mice (binomial tests, FDR-adjusted *P* < 0.05) (table S5). The proportion of ASVs that displayed significantly positive deviations from neutrality in competition experiments was significantly higher for native ASVs than for non-native ASVs (chi-square test, *P* < 0.001), further confirming the local adaptation of native ASVs. Native ASVs favored by selection based on binomial tests (*P* < 0.05) were significantly overrepresented within Bacteroidetes relative to Firmicutes (chi-square test, *P* < 1 × 10^−5^) or to all non-Bacteroidetes lineages (chi-square test, *P* < 1 × 10^−5^) (Fig. 3A). Taxonomic assignments and results of selection analyses for all ASVs are presented in table S5.

Comparing outcomes for native ASVs between WT and *Rag1^−/−^* ex-germ-free mice indicated that, in most cases, selective advantages did not depend on host adaptive immunity (Fig. 3A), consistent with results of β-diversity–based analyses (Fig. 2). However, seven native ASVs were significantly favored by selection in WT hosts (binomial tests, *P* < 0.05) but not in *Rag1*^−/−^ hosts (binomial tests, *P* > 0.05) (Fig. 3, A and B). The negative effects of *Rag1* deletion on ASV-specific selective advantages were only detected within mice that received the FL1 + PAH mixture (i.e., none were detected within mice that received the NY4 + PMAN mixture). Four of seven of these ASVs displayed significantly greater positive deviations in relative abundance from neutrality in WT mice than in *Rag1*^−/−^ mice based on Wilcoxon tests of per-cage mean relative abundances (Benjamin-Hochberg–corrected *P* < 0.05) (Fig. 3C and the Supplementary Materials). Thus, for a subset of native gut bacterial lineages, selective advantages within house mice depended on the presence of *Rag1*, suggesting that fitness advantages for certain native ASVs were mediated by the host adaptive immune system.

We also identified specific non-native ASVs in the FL1 + PAH or NY4 + PMAN mixtures that displayed significant evidence for negative selection in ex-germ-free house mice (table S5). As in the analyses testing for positive selection, these analyses identified multiple ASVs showing evidence of negative selection in ex-germ-free house mice, including ASVs for which negative selection depended on the presence of *Rag1* (fig. S12). Overall, we observed that the deletion of *Rag1* tended to strengthen negative selection on non-native ASVs relative to the WT: 40 ASVs displayed evidence of negative selection in *Rag1*^−/−^ hosts but not in WT hosts, whereas only 9 ASVs displayed evidence of negative selection in WT hosts but not in *Rag1*^−/−^ hosts. Using more stringent criteria for determining host genotype–dependent ASV fitness that incorporated FDR correction, we observed four ASVs belonging to the Bacteroidota for which negative selection significantly differed between WT and *Rag1*^−/−^ hosts (fig. S12C). These results identify specific non-native ASVs under negative selection in house-mouse hosts, including a subset for which negative selection depended on the presence of *Rag1*.

Microbiota competition experiments in germ-free mice, in which microbial dispersal events were controlled, enabled tests for local adaptation of the house-mouse gut microbiota. In all sets of experiments conducted, native microbiotas consistently outcompeted non-native microbiotas within house-mouse hosts. Previous work has shown that, in germ-free house mice, house-mouse microbiotas can outcompete microbiotas from distantly related mammalian host species, such as humans (*38*). Our study shows that house mice microbiotas display competitive advantages over microbiotas from hosts diverged from house mice <10 million years ago, consistent with local adaptation of microbiota over relatively short evolutionary time scales. One limitation of our study was the lack of a non-*domesticus* germ-free mouse model with which to conduct microbiota-competition experiments designed to assess reciprocal home-site advantage of native microbiotas in multiple host species. However, across all experiments, we found that the degree of home-site advantage for native microbiota observed in the competition experiments was modestly but significantly positively associated with the evolutionary divergence times between native and non-native donors whose microbiotas were competed (*P* < 0.01 for non-zero slope; fig. S13). This observation is consistent with the local adaptation of native microbiota to their host-associated environment. The home-site advantage for house-mouse microbiotas did not require the presence of a functional adaptive immune system in hosts, suggesting that other mechanisms, such as innate immunity (*39*), host glycan structure (*40*), or microbe-microbe interactions, may mediate the local adaptation of native gut microbiota. These results demonstrate natural selection favoring the assembly of host species–specific mammalian gut microbiota.

## MATERIALS AND METHODS

### Ethical statement

All procedures conformed to guidelines established by the U.S. National Institutes of Health and have been approved by the Cornell University Institutional Animal Care and Use Committee (protocol #2015-0060). The Institutional Animal Care and Use Committee at the University of Nebraska-Lincoln approved all procedures involving germ-free and ex-germ-free mice (protocol #1700).

### Animal husbandry

Donor fecal samples were collected from five lines of *M. m. domesticus* (NY2, NY4, FL1, LEWES/EiJ, and C57BL/6 J), two lines of *M. spicilegus* (ZBN and SPI/TUA), one strain of *M. pahari* (PAHARI/EiJ), and one line of *P. maniculatus* (referred to here as PMAN). All *Mus* lines were maintained by sibling mating in a common laboratory environment using standard mouse husbandry procedures for at least 20 generations. Wild-derived inbred *M. m. domesticus* lines NY2 and NY4 were derived from distinct initial pairings of wild mice trapped from Saratoga Springs, NY, USA, whereas the FL1 line was derived from a pair of wild mice trapped in Gainsville, FL, USA. Additional house-mouse strains of *M. m. domesticus* (LEWES/EiJ and C57BL/6 J), *M. spicilegus* (ZBN and SPI/TUA), and *M. pahari* (PAHARI/EiJ) lines were originally obtained from the Jackson Laboratory (Bar Harbor, Maine, USA), where the lines were maintained without rederivation. These lines from the Jackson Laboratory were subsequently maintained at Cornell University alongside the *domesticus* lines for >10 generations. The *P. maniculatus* line was originally derived by the Peromyscus Genetic Stock Center (University of South Carolina, Columbia, SC, USA). All mice were fed standard laboratory mouse chow (~19% crude protein, ~6% fat, 44% carbohydrate, and ~18% fiber). Fecal samples from individual mice were weighed and combined into mixtures at Cornell University and then shipped on dry ice overnight to the University of Nebraska-Lincoln (details in table S6). Our previous work has shown that >85% of the family-level taxonomic diversity in mouse gut microbiota remains viable after freezing (i.e., >85% of the bacterial families detected in fecal samples that we cultivated under anaerobic conditions on a panel of nine media) (*41*). Fecal slurries were suspended in reduced, sterile 50 μl of phosphate-buffered saline and then 10 μl of phosphate-buffered saline gavaged into individual germ-free mice.

Germ-free recipient *M. m. domesticus* (C57BL/6J and *Rag1*^−/−^) were born and reared in flexible film isolators and maintained under gnotobiotic conditions (temperature of 20°C, relative humidity of 60%, and 14-hour light/10-hour dark cycle) by the Nebraska Gnotobiotic Mouse Program at the University of Nebraska-Lincoln. For experiment 1 (i.e., experiment shown in Fig. 1C), male and female germ-free mice were transferred from isolators to sterile, individually ventilated cages with high-performance filter lids at the time of colonization. For experiment 2 (i.e., experiment shown in Fig. 2A), germ-free mice were colonized and maintained in cages within gnotobiotic isolators for the duration of the study. In addition, all WT and Rag1^−/−^ mice were raised and maintained in the same gnotobiotic facility and were age- and sex-matched. All mice for both experiments were fed autoclaved chow (LabDiet 5K67, Purina Foods, St. Louis, MO, USA) ad libitum.

### 16*S* rRNA amplicon profiling

DNA was extracted from all samples using the DNeasy PowerLyzer PowerSoil Kit from QIAGEN (Hilden, Germany). Donor fecal samples, inoculum mixtures, and output fecal samples from ex-germ-free mice used in experiment 1 were sent to the Microbiome Core Lab located at the Jill Roberts Institute for Research in Inflammatory Bowel Disease of Weill Cornell Medicine (New York, NY, USA) for sequencing. Briefly, 16*S* rRNA (V4-V5) amplicon libraries were prepared using 515F-926R primers developed by the Earth Microbiome Project (*42*). Library pools were then sequenced in one PE250 run on an Illumina MiSeq (San Diego, CA, USA).

DNA from donor fecal samples and output fecal samples from ex-germ-free mice used in experiment 2 were sent to the Integrated Microbiome Resource at Dalhousie University (Halifax NS, Canada). 16*S* rRNA (V4-V5) amplicon libraries were prepared using 515F-926R primers. Libraries were sequenced in a single PE300 Illumina MiSeq run with a V3 reagent chemistry (San Diego, CA, USA).

Raw reads from both experiments were denoised into ASVs using the DADA2 pipeline (v1.14.0) (*43*). Following generation of an ASV table, sequences were chimera-checked, and those remaining that were not 230 to 235 base pairs in length were removed. Taxonomic assignments were made using the RDP classifier and both GreenGene database (13_8) (*44*) and the SILVA nr database (v132) (*45*) (table S5). ASVs that were not identified as Domain Bacteria or Domain Archaea were excluded from further analyses. A phylogenetic tree of the total set of ASVs was inferred using the fragment insertion function (*46*, *47*) in QIIME2 (v2019.1) (*48*) and the full GreenGenes 13_8 tree. A mapping file, the ASV table, the taxonomy table, and the phylogenetic tree were imported into R and combined into a single phyloseq object (v1.28.0) (*49*).

### 16*S* rRNA gene copy number quantification

The Femto Bacterial DNA Quantification Kit (Zymo, Irvine, CA, USA) was used to quantify the bacterial load of donor and ex-germ-free mouse fecal samples on the Applied Biosystems QuantStudio 7 Pro Real-Time PCR System (Waltham, MA, USA). Extracted DNA was diluted and quantified using the standard analysis protocol. Technical replicates (i.e., replicates of the same DNA sample) were averaged.

### Data analysis

Shannon diversity index and the Jaccard similarity index were calculated performed using the phyloseq R package. Pielou’s evenness index was calculated using the microbiome R package (v1.16.0). Additional statistical analyses including chi-square, Fisher’s exact, exact binomial, and Wilcoxon tests were performed in R. PERMANOVA tests were performed using the “adonis” function in the vegan R package (v2.5.5). PCoA ordinations, boxplots, and scatter plots were generated using the ggplot2 package in R (v3.2.0). Phylogenetic trees were created using the iTOL online tool (v6) (*50*).

### LAI calculations

To assess the degree of local adaptation of microbiota to house mice, we defined the LAI as the Jaccard similarity of the microbiota of the ex-germ-free mouse to that of the native house-mouse donor minus the similarity of the microbiota of the ex-germ-free mouse to that of the non-native donor.

$$\begin{array}{c} \text{Local adaptation index}\;(A)\\ =\left(1-\frac{|A\cap {\mathrm{Donor}}_{\mathrm{Native}}|}{|A\cup {\mathrm{Donor}}_{\mathrm{Native}}|}\;\right)-\left(1-\frac{|A\cap {\mathrm{Donor}}_{\mathrm{Non}-\mathrm{native}}|}{|A\cup {\mathrm{Donor}}_{\mathrm{Non}-\mathrm{native}}|}\right) \end{array}$$

Larger positive LAI values indicate that a given profile from ex-germ-free mice was more similar to its native donor than it was to its non-native donor, indicative of local adaptation of the gut microbiota to the *M. m. domesticus* gut. The LAI was also calculated for each microbiota simulated under a neutral model of community assembly. These values were used as the expected LAI values for ex-germ-free mice given neutral assembly. Therefore, the difference between the observed LAI for an ex-germ-free mouse fecal microbiota and the expected LAI for the microbiota (i.e., observed LAI–expected LAI) provided a test statistic for the degree of competitive advantage of the native microbiota over the non-native microbiota. For this and later LAI calculations, we focus on the ASV-level analyses (i.e., the finest scale afforded by the data) because it provides the greatest resolution to differentiate between native and non-native microbiotas. LAI values were calculated independently for each sampling time point.

### Simulation of expected composition under neutral assembly

We simulated the microbiota composition expected under a model of neutral community assembly for each ex-germ-free mouse inoculated with a mixture of native and non-native microbiotas. For these analyses, we incorporated both the compositions of the donor samples estimated from 16*S* rRNA gene sequencing and the bacterial loads of the donor samples estimated from quantitative polymerase chain reaction (qPCR) of the 16*S* rRNA gene. We measured the bacterial load of each donor sample using qPCR and multiplied the copies per gram by the mass of each fecal pellet included in the inoculum mixture to yield an estimate of the total number of 16*S* rRNA gene copies in the mixture from each donor. The fractional proportion of each donor’s microbial load was then used to weight the subsampling of the microbiotas from each donor strain to generate the expected microbiota composition in the ex-germ-free mouse under neutral assembly. For analyses of selection on individual ASVs, the expected relative abundance of each ASV for each mixture was calculated on the basis of neutral assembly using the weightings from the observed 16*S* rRNA gene profiles and loads in the donor fecal samples. Further details of these analyses, as well as a fully reproducible workflow, are available as a Supplementary Code file.

### SourceTracker analysis

SourceTracker (v1.0.1) (*29*), a Bayesian method for estimating the likelihood of microbes within a sample having originated from each of multiple sources, was used to assess the most likely origin of ASVs in each fecal sample from ex-germ-free mice. Here, we estimated the most likely origin of ASVs in every fecal pellet from ex-germ-free mice using the corresponding native and non-native donors as the “source” communities. Sample and donor communities were rarefied to the same, minimum sequencing depth before SourceTracking to control for differences in sequencing depth between donors. SourceTracker was used with default settings.

### Differential abundance analysis

To identify ASVs that consistently differentiated native and non-native donors, we tested for ASVs displaying differential abundance between native and non-native donors using DESeq2 (*51*) using default settings. These analyses focused only ASVs present in three or more native donors or three or more non-native donors.

## Supplementary Materials

### This PDF file includes:

Supplementary Results Figs. S1 to S13 Legends for tables S1 to S6 Legend for data S1
