# Supplementary material for: Home-site advantage for host species–specific gut microbiota
Source: Sci Adv. 2023 May 12;9(19):eadf5499. doi: 10.1126/sciadv.adf5499 (PMC10184861; doi:10.1126/sciadv.adf5499)
Supplement: Supplementary file 1 — Supplementary Results Figs. S1 to S13 Legends for tables S1 to S6 Legend for data S1 [file sciadv.adf5499_sm.pdf]

Science Advances
AAAS

Supplementary Materials for

Home-site advantage for host species–specific gut microbiota

Daniel D. Sprockett *et al.*

Corresponding author: Andrew H. Moeller, andrew.moeller@cornell.edu

Sci. Adv. **9**, eadf5499 (2023)
DOI: 10.1126/sciadv.adf5499

The PDF file includes:

Supplementary Results
Figs. S1 to S13
Legends for tables S1 to S6
Legend for data S1

Other Supplementary Material for this manuscript includes the following:

Tables S1 to S6
Data S1

Supplementary Results

Ordination plots

The ordination plots presented in Fig 1D and 2B were calculated using the Jaccard similarity index followed by a Principal Coordinate Analysis (PCoA). For both sets of experiments, a single plot was faceted by inoculum to increase the visual clarity with which to assess the relationship between each ex-germ-free mouse output microbiota to their two constituent donors.

Singly-Colonized Mouse Experiments

In addition to experiments in which germ-free mice were colonized with mixtures of native and non-native microbiotas, we also conducted experiments in which germ-free mice were colonized by fecal microbiotas from a single mouse lineage. These experiments were not conducted with sufficient replication to provide power to assess pairwise differences in colonization success in the single inoculed mice between lines; however, these experiments did allow tests of whether there colonization success significantly differed between native and non-native donors (as groups). These data showed that many non-native microbes not observed in microbiota competition experiments were able to colonize the germ-free mouse gut when single microbiota inoculations were performed. Although many of these non-native ASVs were not observed in the ex-germ-free mice in which competition experiments between native and non-native microbiota were conducted, the observation that these ASVs were able to colonize germ-free house mice inoculated with a single microbiota demonstrates that the house-mouse gut represents potential niche space for these non-native ASVs. An ASV table on which all analyses were based that includes a list of all ASVs detected in non-native donors and singly colonized mice but not mice colonized with microbiota mixtures is presented in Additional Data File 1.

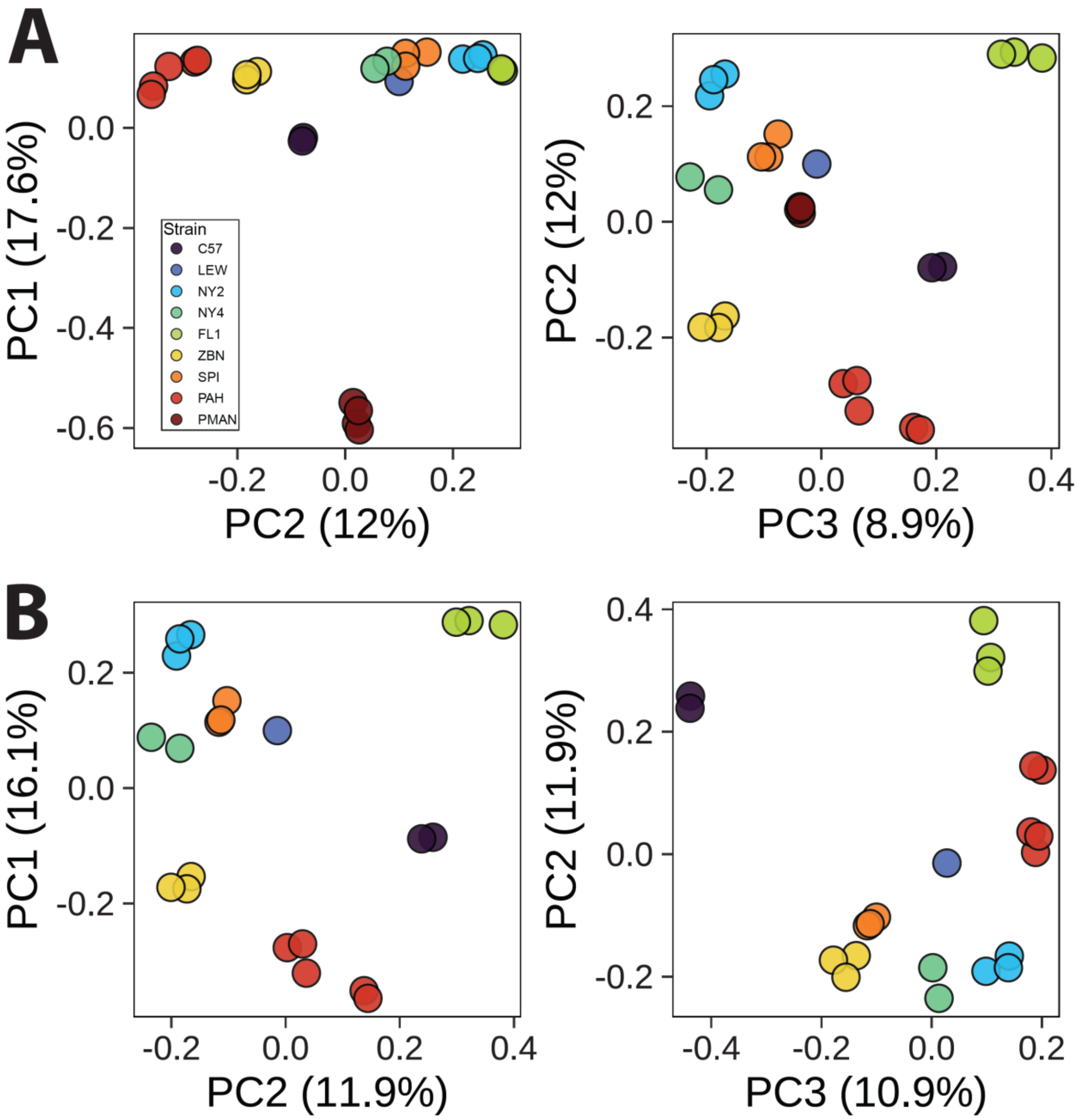

Fig. S1. Microbiotas from diverse mouse lineages were compositionally distinct. (**A, B**) Principal Coordinate (PC) Analysis plots show similarity of microbiotas among wild-derived mouse lineages based on the Jaccard similarity index of 16S rRNA amplicon profiles generated from donor fecal samples (i.e., fecal samples gavaged into germ-free mice). Plots show all donor

mouse samples used in this study (**A**) or only donor samples from *Mus* (**B**). Colors indicated the rodent strain from which the fecal sample originated. PERMANOVA $p < 0.001$ for A and B.

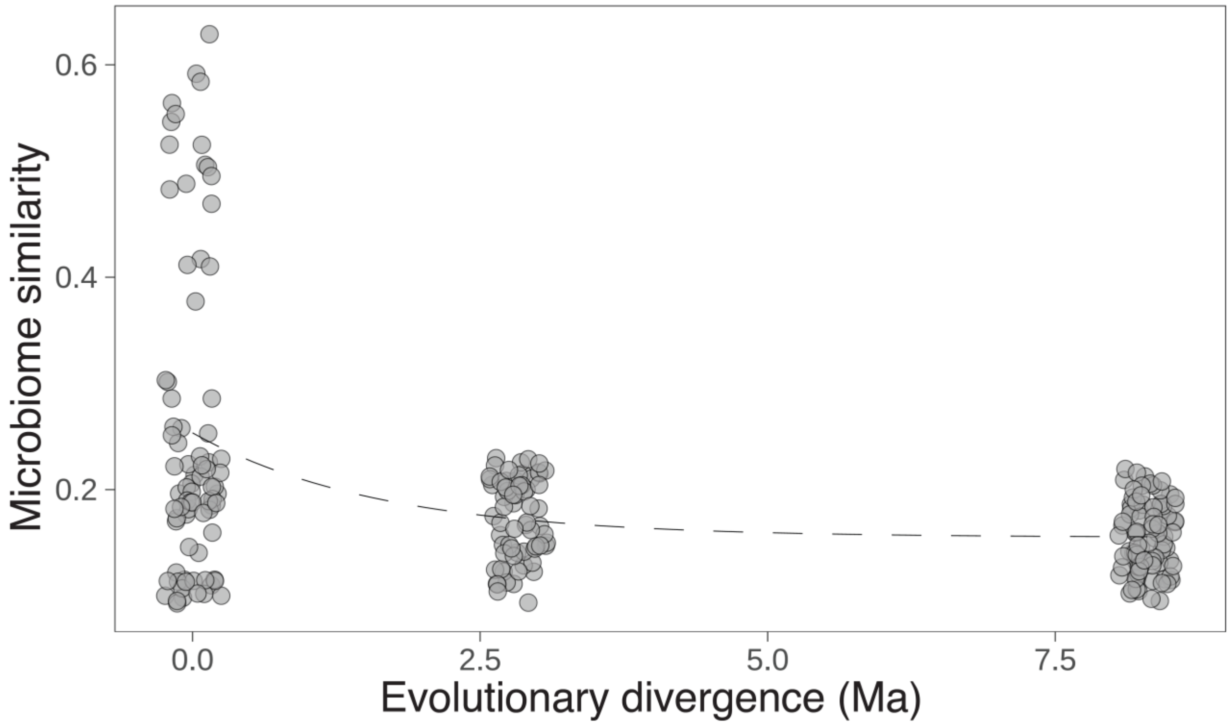

Fig. S2. Microbiotas from *Mus* lines recapitulated donor phylogeny. Scatterplot shows the negative relationship between microbiota similarity and host evolutionary divergence time (Ma, millions of years ago) among different mouse lines in the genus *Mus* based on the Jaccard similarity index. Dashed line indicates exponential decay ($p < 0.001$, $R^2 = 0.19$). Boxplots in inset show Jaccard similarities between pairs of samples from the same host strain (leftmost), different host strains from the same species (middle), and different *Mus* species (right). For each boxplot, the center line denotes the median, and the lower and upper hinges correspond to the first and third quartiles, respectively. Wilcoxon tests, FDR-adjusted p-values **** < 0.0001.

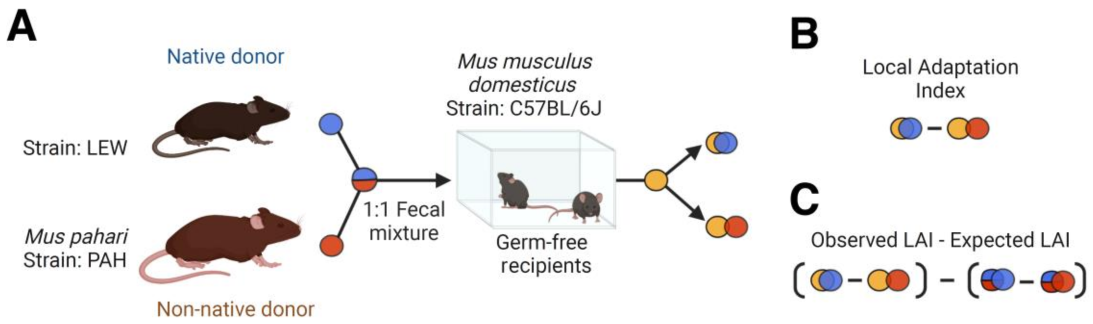

Fig. S3. Calculation of Local Adaptation Index. (**A**) Fecal pellets from native and non-native donors were mixed 1:1 by weight and then gavaged into germ free mice. (**B**) Microbiota similarities between the microbiotas of ex-germ-free mice and donors were calculated. The Local Adaptation Index of a microbiota of an ex-germ-free mouse was defined as the dissimilarity of the microbiota to the native donor minus the dissimilarity of the microbiota to the non-native donor. (**C**) Calculating the difference between the observed LAI and the expected LAI under neutrality (i.e., even mixtures of native and non-native microbiota, accounting for variation in microbial load and weight of donor fecal pellets) provided a test statistic for local adaptation.

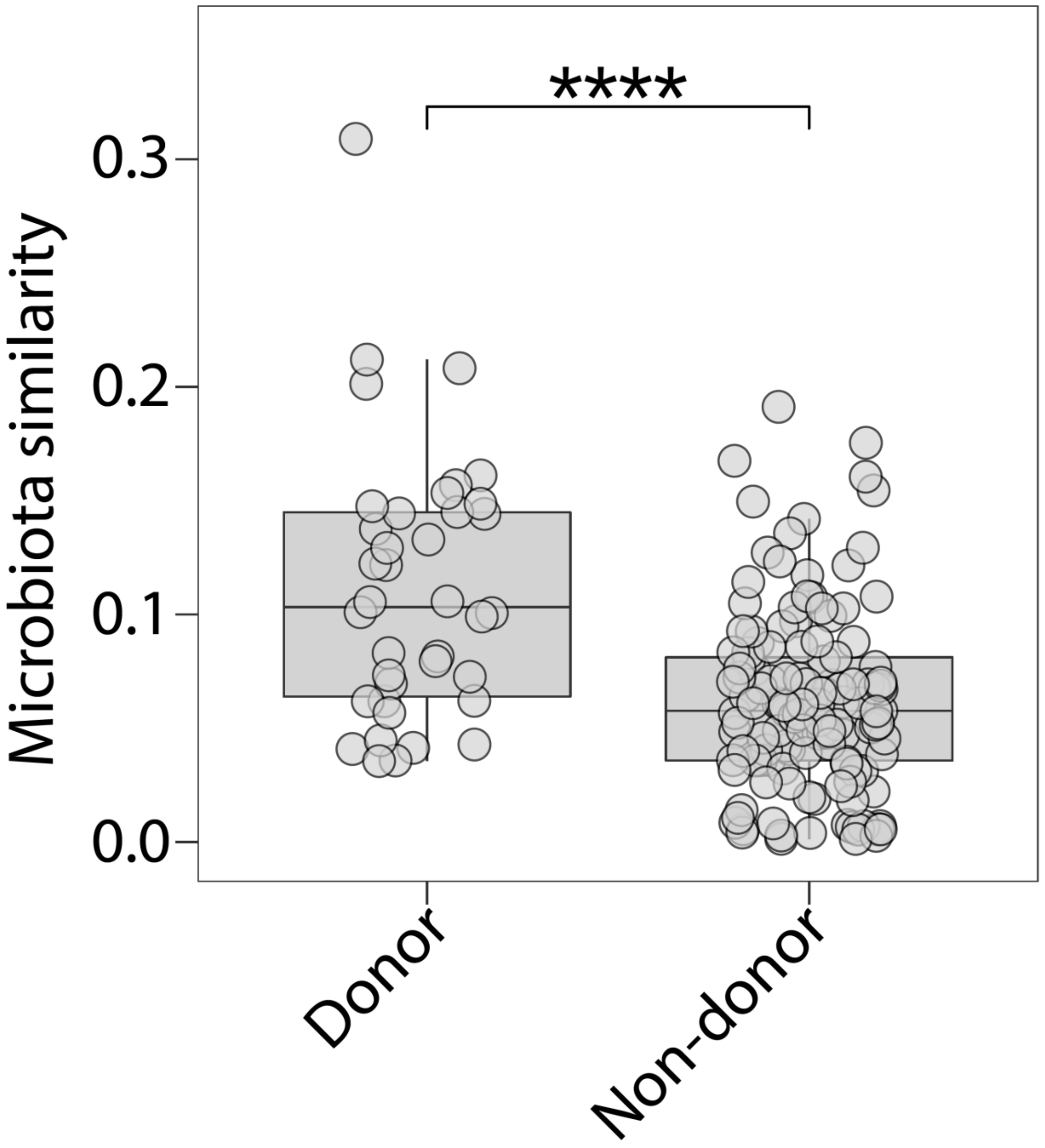

Fig. S4. Microbiota similarity of ex-germ-free mice to donor mouse fecal samples. Boxplots show, for experiment 1 (Fig. 1), the Jaccard similarity between ex-germ free mice and their corresponding donors as well as the Jaccard similarity between ex-germ free mice and other donors. Significance of Wilcoxon tests is indicated by asterisk; **** p-value < 0.0001.

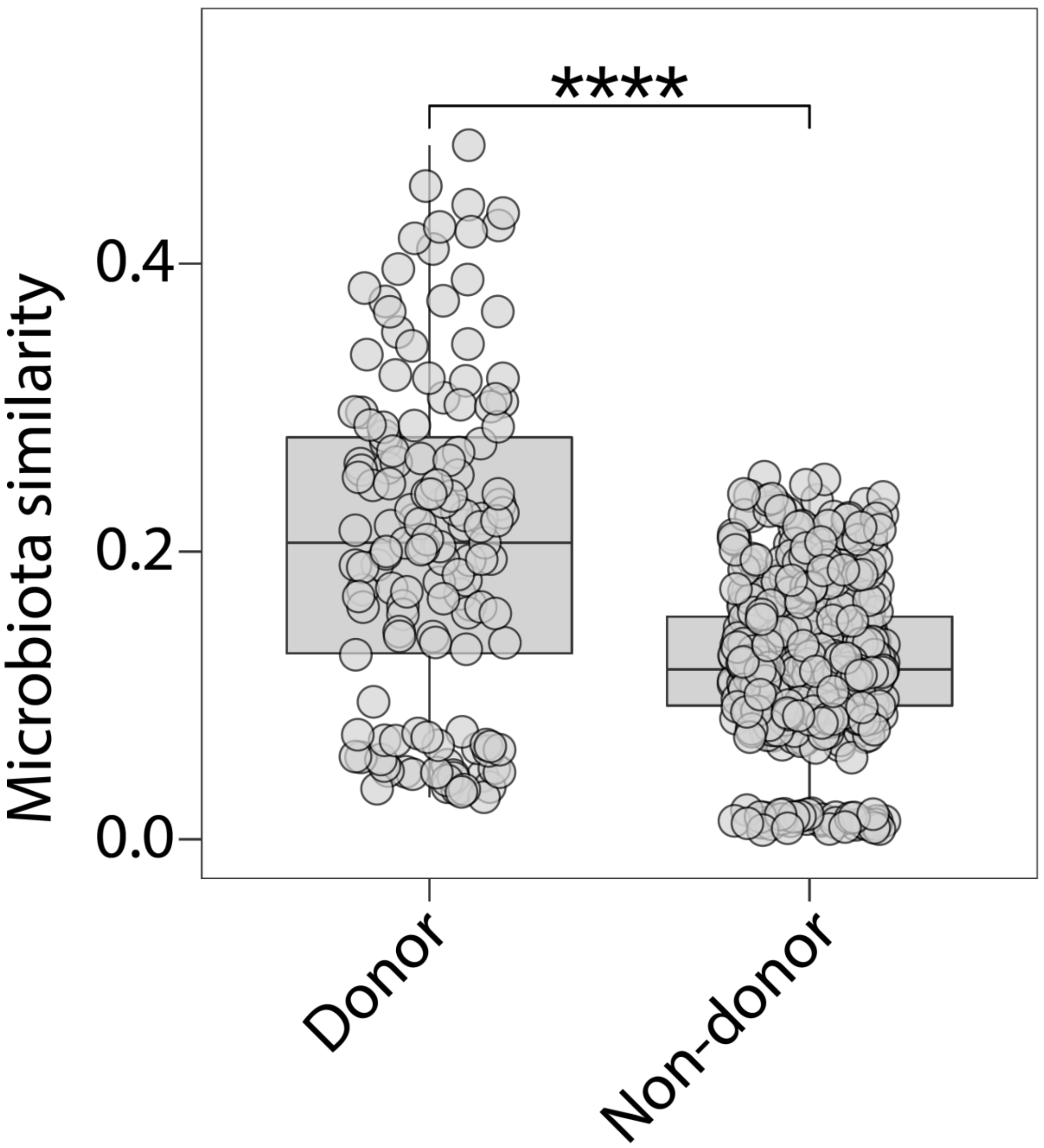

Fig. S5. Microbiota similarity of ex-germ-free mice to donor mouse fecal samples. Boxplots show, for experiment 2 (Fig. 2), the Jaccard similarity between ex-germ free mice and their corresponding donors as well as the Jaccard similarity between ex-germ free mice and other donors. Significance of Wilcoxon tests is indicated by asterisk; **** *p*-value < 0.0001.

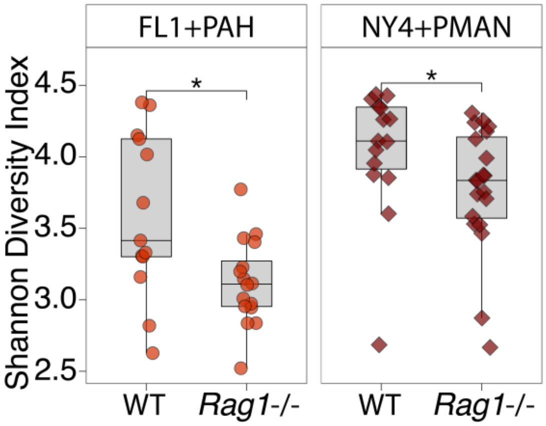

Fig. S6. Alpha diversity differs between WT and *Rag1*$^{-/-}$. Boxplots show, for experiment 2 (Fig. 2), the alpha diversity (Shannon) of ex-germ free mice. Significance of Wilcoxon tests is indicated by asterisk; **** *p*-value < 0.0001.

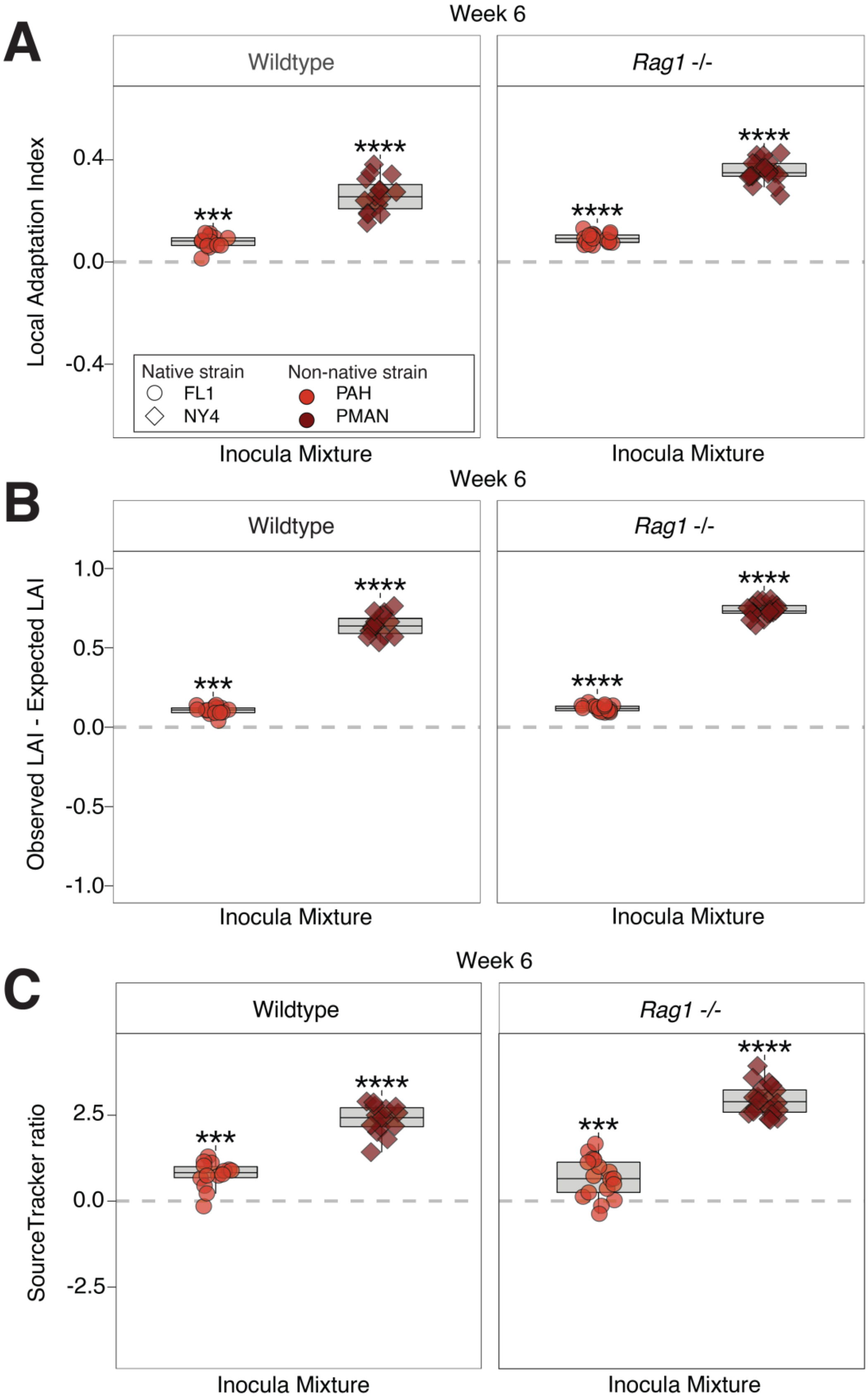

Week 6
A
Wildtype
Rag1 -/-
Local Adaptation Index
0.4
0.0
-0.4
***
****
****
****
Native strain
FL1
NY4
Non-native strain
PAH
PMAN
Inocula Mixture
Inocula Mixture
Week 6
B
Wildtype
Rag1 -/-
Observed LAI - Expected LAI
1.0
0.5
0.0
-0.5
-1.0
***
****
****
****
Inocula Mixture
Inocula Mixture
Week 6
C
Wildtype
Rag1 -/-
SourceTracker ratio
2.5
0.0
-2.5
***
****
***
****
Inocula Mixture
Inocula Mixture

Fig. S7. Adaptive advantages of native over non-native microbiotas persisted to week 6 in both WT and *Rag1*$^{-/-}$ mice. (**A**) Boxplots show positive Local Adaptation Index (LAI) values, indicating that microbiotas of ex-germ-free WT and *Rag1*$^{-/-}$ mice at week 6 were more similar to the microbiotas of their native donors than they were to those of their non-native donors. (**B**) Boxplots show positive differences between observed LAI values and LAI values expected under neutral assembly. (**C**) Boxplots show positive $\log_{10}$ transformed ratios between the percentage of the microbiota derived from the native donor (estimated with SourceTracker) to the percentage derived from the non-native donor. Shapes and colors denote identities of native and non-native donors, respectively. Lines connect samples from the same mouse. Wilcoxon test for non-zero mean, FDR-adjusted p-values * < 0.05, ** < 0.01, *** < 0.001, **** < 0.0001. For each boxplot in (**A**–**C**), the center line denotes median, and lower and upper hinges correspond to first and third quartiles, respectively.

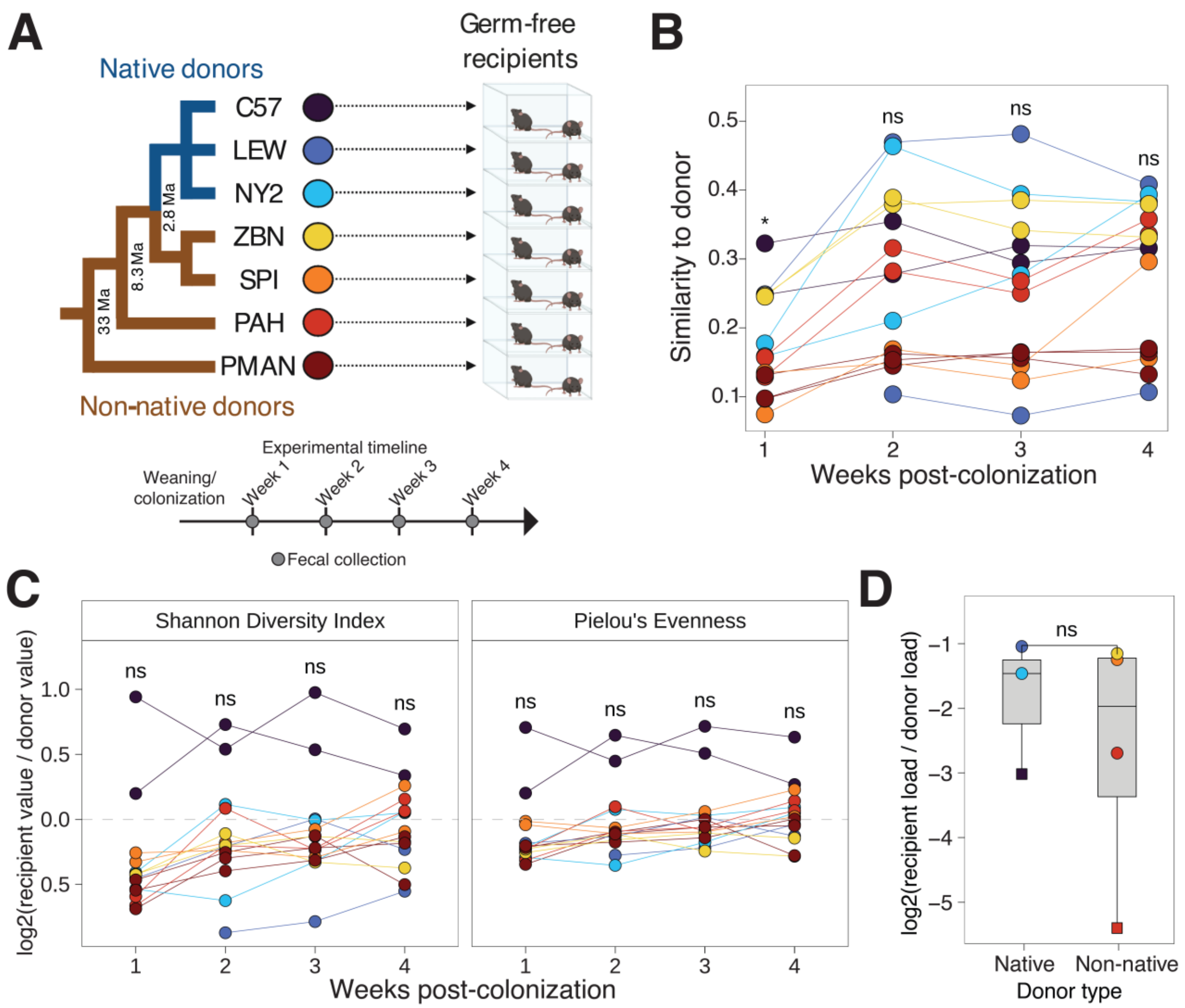

Figure S8. Native and non-native microbiotas colonized germ-free mice to comparable degrees when inoculated individually. (**A**) Cartoons show experimental design. Fecal pellets from three native *Mus musculus domesticus* lines and four non-native mouse strains were inoculated directly into germ-free mice. Fecal pellets were collected from ex-germ-free mice weekly for 4 weeks. (**B**) Lines show Jaccard distanced of microbiotas from ex-germ-free mice to their donor microbiotas over the 4-week experiment. Each line corresponds to an ex-germ-free mouse, and each point the compositionally dissimilarity between the microbiota from an ex-germ-free mouse and the donor microbiota that the mouse received. (**C**) Lines show $\log_2$-transformed ratios of Shannon's Diversity Index and Pielou's Evenness between the microbiotas

of ex-germ-free mice and their donor microbiotas during the 4-week experiment. (**D**) Boxplots show log_2-transformed ratio between the bacterial loads per gram of feces in ex-germ-free recipients and donors. Ex-germ-free mice that received house-mouse microbiota are plotted separately from those that received non-native microbiotas. For panels (**A**–**D**), colors denote the identities of the mouse donors. Lines connect samples from the same mouse. Wilcoxon test, FDR-adjusted p-values ns = not significant, ns = not significant, * < 0.05.

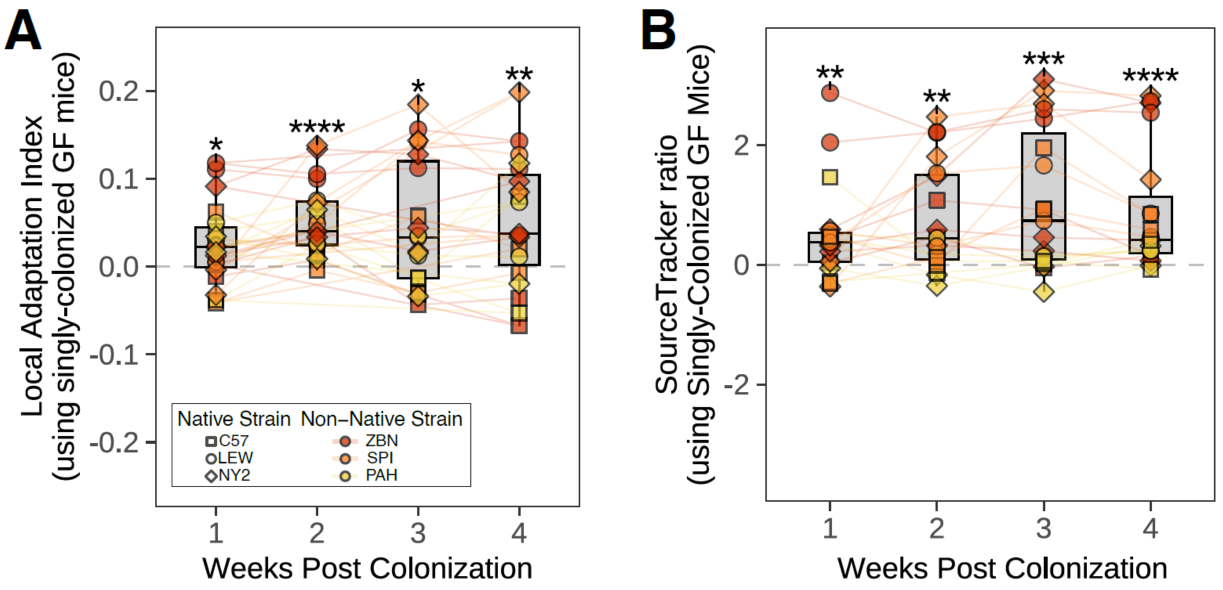

Figure S9. Modified local adaptation index for experiment shown in Fig. 1, considering only the ASVs that were observed in germ-free mice gavaged with a single native or non-native microbiota. Boxplots show positive (**A**) Local Adaptation Index (LAI) values and (**B**) SourceTracker ratios throughout the 4-week experiment of ex-germ-free mice. For (**A**) and (**B**), LAI was calculated based on an ASV table containing only ASVs detected in mice gavaged with a single microbiota (Fig. S8). Only ex-germ-free mice from microbiota competition experiments for which both donor microbiotas were also gavaged individually into germ-free mice individually are shown. FDR-adjusted *p*-values were derived from Wilcoxon tests for non-zero mean, * < 0.05, ** < 0.01, *** < 0.001 **** < 0.0001.

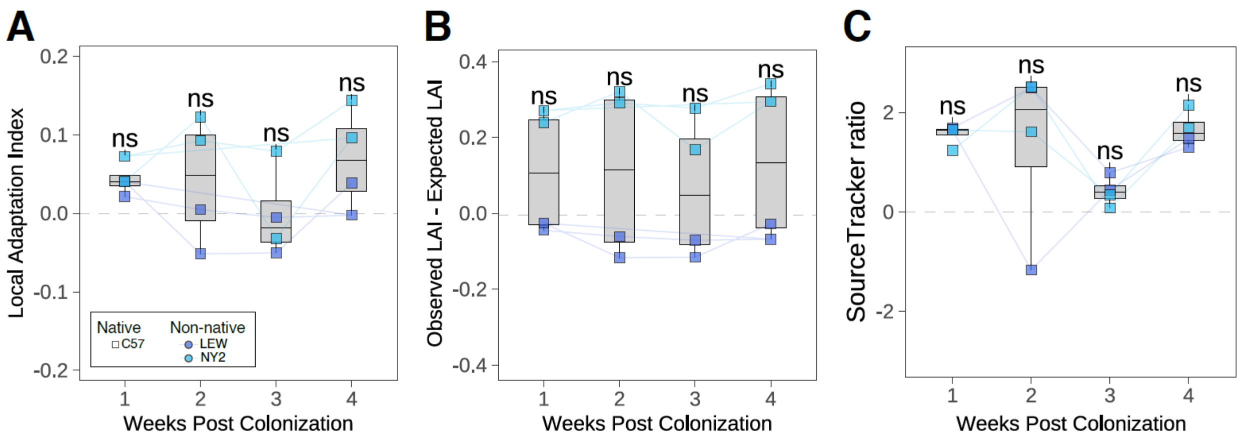

Figure S10. Non-significant trend towards home-site advantage of C57 microbiota over wild-derived *domesticus* microbiota. (**A**) Boxplots show Local Adaptation Index (LAI) values of ex-germ-free mice throughout the 4-week experiment; (**B**) positive differences between observed LAI values and LAI values expected under neutral assembly; and (**C**) log_{10} transformed ratios of native ASVs to non-native ASVs identified as sources by SourceTracker. In (**A**–**C**), shapes and colors denote identities of native and non-native donors, respectively, as indicated by the key in (**A**). Lines connect samples from the same mouse. For each boxplot in (**A**–**C**), center lines denote medians, and lower and upper hinges correspond to first and third quartiles, respectively. FDR-adjusted *p*-values were derived from Wilcoxon tests for non-zero mean, ns > 0.05.

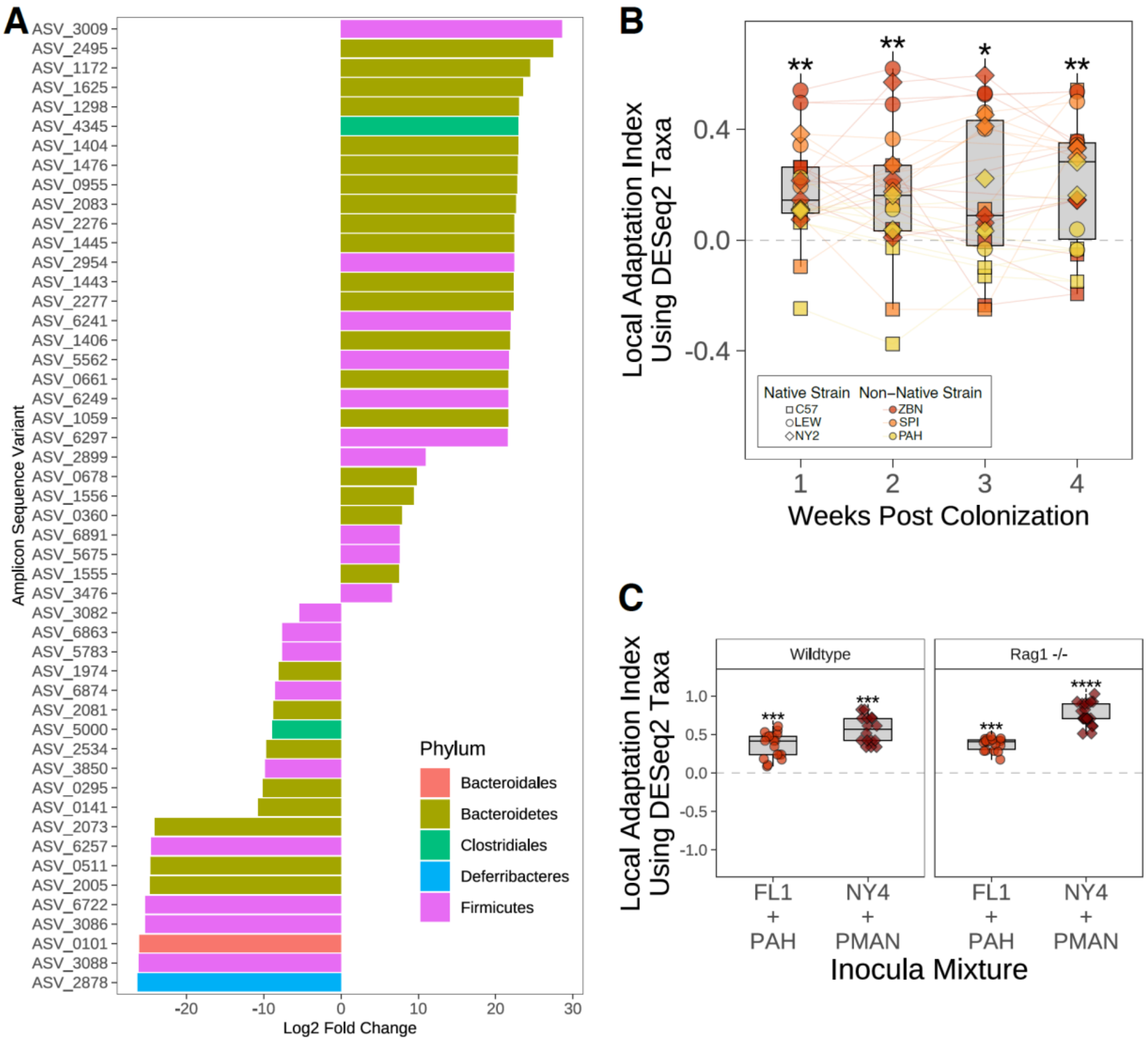

Figure S11. ASVs that were differentially abundant between native and non-native donors also displayed positive LAI values in ex-germ-free mice. (**A**) Bars indicated differentially abundant ASVs between native (positive values) and non-native (negative values) donors. All ASVs were present in at least three native donors or at least three non-native donors. Bars represents ASVs and are colored by phylum. (**B**) Boxplots show Local Adaptation Index (LAI) values of differentially abundant ASVs in ex-germ-free mice throughout the first experiment (Fig. 1). (**C**) Boxplots show Local Adaptation Index (LAI) values of differentially abundant ASVs in ex-germ-free mice at week 4 in the second experiment (Fig. 2). FDR-adjusted *p*-values

were derived from Wilcoxon tests for non-zero mean, * < 0.05, ** < 0.01, *** < 0.001, **** < 0.0001.

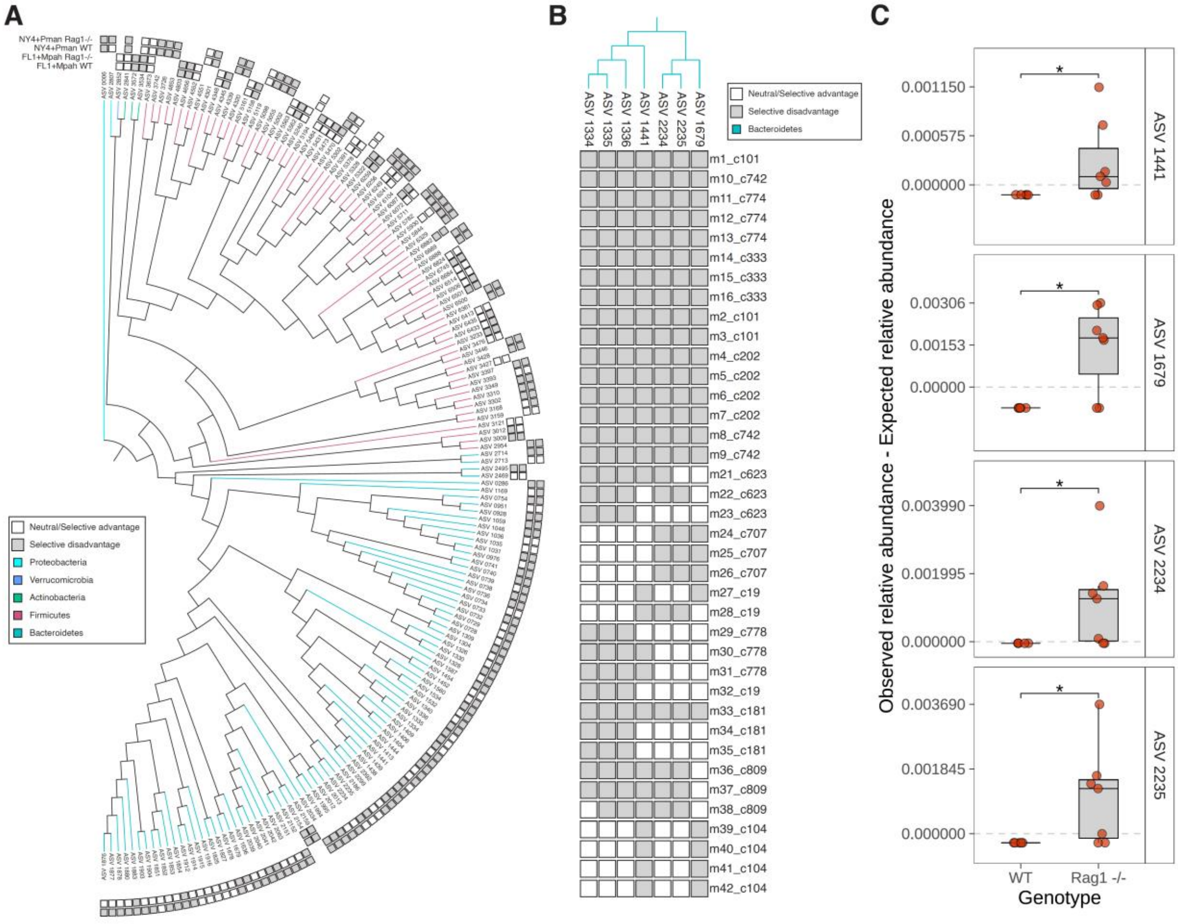

Figure S12. Selective disadvantages for a subset of non-native ASVs depended on *Rag1*. (**A**) Phylogeny shows relationships among non-*domesticus*-specific ASVs (i.e., ASVs restricted to non-native donors) detected in ex-germ-free mice that received the NY4+PMAN or FL1+PAH microbiota mixtures. Colors of branches denote bacterial phyla. Rings correspond to ex-germ-free mouse groups and indicate significant negative selection on ASVs (filled squares) within ex-germ-free mice based on binomial tests. Unfilled squares mark ASVs that were detected in ex-germ-free mice but not significantly negatively selected. Absence of squares indicates that the ASV was not detected in the mouse group. (**B**) Phylogeny from (**A**) pruned to only ASVs in the NY4+PMAN mixture displaying significant selective disadvantages in WT ex-germ-free mice but not in *Rag1*$^{-/-}$ mice. Rows correspond to individual ex-germ-free mice, and columns

correspond to the tips of the phylogeny. Filled squares indicate ex-germ-free mice in which the observed relative abundance of the ASV was observed to be less than the relative abundance expected under neutrality. (**C**) Boxplots display differences between observed and expected ASV relative abundances in WT and *Rag1*$^{-/-}$ mice that received the NY4+PMAN mixture. All ASVs from both inocula in (**B**) for which cage-mean differences between host genotypes remained significant after false discovery rate (FDR) correction are shown. FDR-adjusted p-values * < 0.05, ** < 0.01.

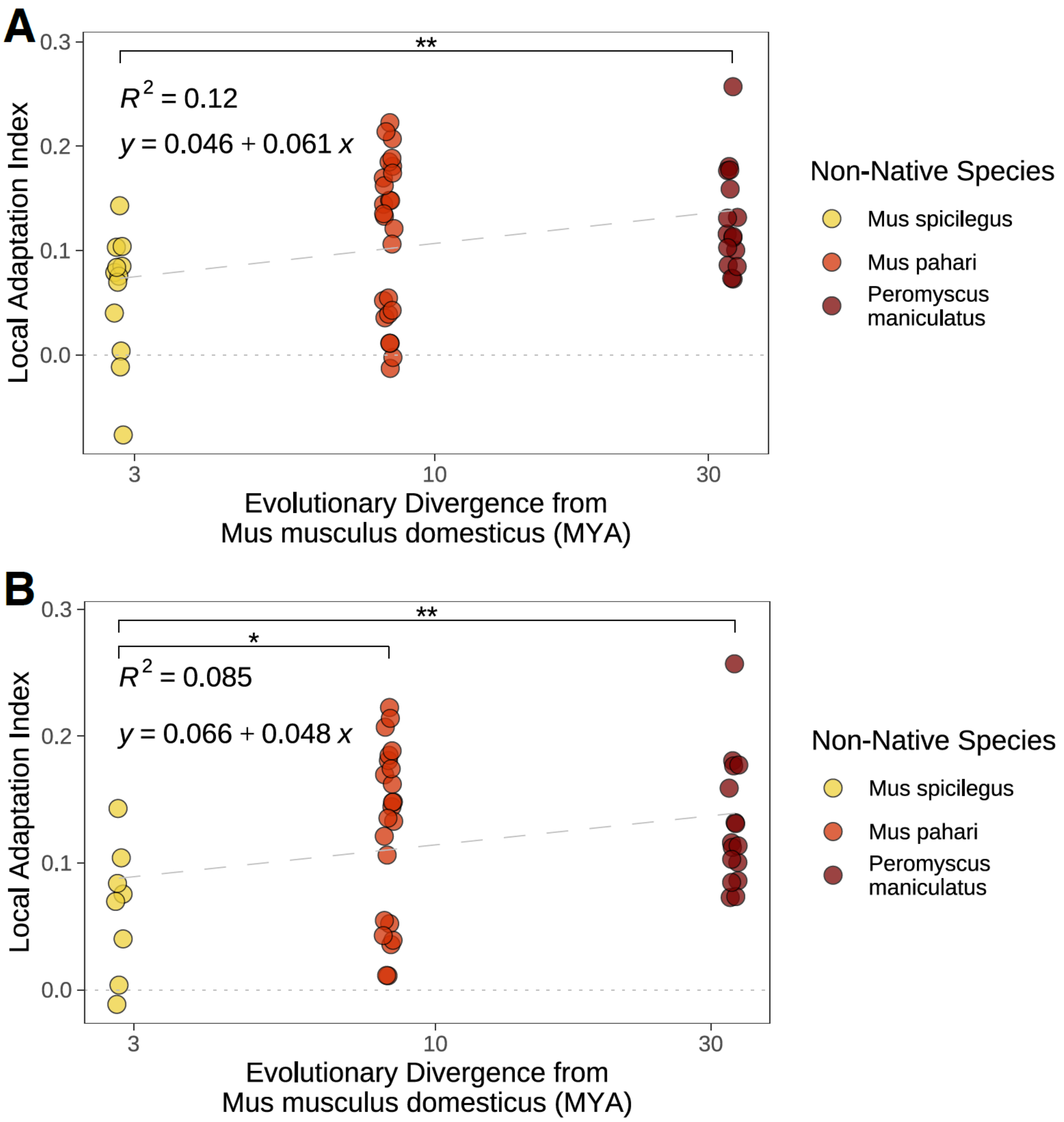

Figure S13. Association of LAI and evolutionary divergence between native and non-native donors. Regression analyses in (**A**) and (**B**) show the relationship between LAI (at week 4) and the evolutionary divergence between native and non-native donors. Plot in (A) shows relationship with all data included, and plot in (B) shows data only mice that received microbiota

mixtures from wild-derived mouse lines (i.e., microbiota mixtures without C57 microbiota).

FDR-adjusted *p*-values were derived from Wilcoxon tests, * < 0.05, ** < 0.01.

Table S1. Metadata for rodent donor and recipient samples.

Table S2. Local Adaptation Index values from experiment 1.

Table S3. Bacterial load estimates using 16S rRNA gene targeted qPCR.

Table S4. Local Adaptation Index values from experiment 2.

Table S5. Selection results from NY4+Pman inoculum.

Table S6. Preparations of mixed inocula

Supplementary Data File 1. ASV table generated from all experiments.
